# Focal inputs are a potential origin of local field potential (LFP) in the brain regions without laminar structure

**DOI:** 10.1371/journal.pone.0226028

**Published:** 2019-12-11

**Authors:** Takuma Tanaka, Kouichi C. Nakamura

**Affiliations:** 1 Graduate School of Data Science, Shiga University, Hikone, Shiga, Japan; 2 MRC Brain Network Dynamics Unit, University of Oxford, Oxford, United Kingdom; Sorbonne Universite UFR de Biologie, FRANCE

## Abstract

Current sinks and sources spatially separated between the apical and basal dendrites have been believed to be essential in generating local field potentials (LFPs). According to this theory, LFPs would not be large enough to be observed in the regions without laminar structures, such as striatum and thalamus. However, LFPs are experimentally recorded in these regions. We hypothesized that focal excitatory input induces a concentric current sink and source generating LFPs in these regions. In this study, we tested this hypothesis by the numerical simulations of multicompartment neuron models and the analysis of simplified models. Both confirmed that focal excitatory input can generate LFPs on the order of 0.1 mV in a region without laminar structures. The present results suggest that LFPs in subcortical nuclei indicate localized excitatory input.

## Introduction

**Fig 1:**
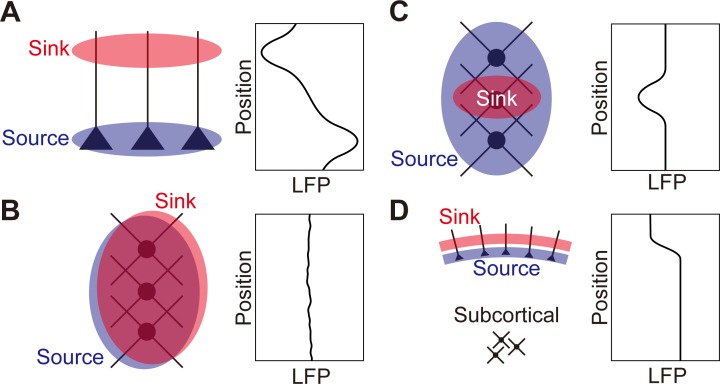
Hypotheses on LFP generation. Schematics of sink and source arrangements (left) and expected LFPs (right). (A) In the cortex, a current sink (red) on apical dendrites and a current source (blue) on basal dendrites generate large LFPs. (B) Overlapping current sink (red) and source (blue) in the non-laminar structures, e.g. the striatum, cannot generate large enough LFPs to be observed. (C) In the striatum, if a focus of excitatory input is formed, the focus serves as a current sink (red), which mostly overlaps with a current source (blue). At the periphery of the focal excitatory input, however, dendrites that are not receiving excitatory input serve as the current source that is not canceled out by the current sink. (D) Spatially extended synchronization of cortical pyramidal neurons forms sheets of current sink (red) and source (blue) that generate the subcortical LFPs of roughly the same magnitude and polarity as the deep cortical layers.

Local field potentials (LFPs) have been reported not only in the neocortex [1] but also in the subcortical structures such as the striatum [2–5], thalamus [6–9], and other regions including the basal ganglia [10,11]. LFPs have been used to obtain information about sensory or cognitive processes that cannot be obtained by spikes only. The LFPs arise from transmembrane currents of neurons [1,12,13]. In the cortex, contributions from synaptic currents have been believed to be the dominant factor underlying LFPs. The excitatory synaptic currents on the apical dendrites (current sink) and resulting return currents on the basal dendrites (current source) of cortical pyramidal neurons are separated in space. LFPs are a linear superposition of the electric potentials that are generated by these separated sinks and sources (Fig 1A). In contrast, stellate neurons in layer IV have been believed to contribute little to cortical LFPs because the sink and source would cancel out due to the randomly radiating dendritic arbors (Fig 1B) [1]. Likewise, LFPs would not be observed in the striatum or thalamus, which lack laminar structures or polarized dendritic arrangement of the constituent neurons. There might be a local imbalance between the sink and source, which could generate electric potentials [1], but it has not been examined whether LFPs generated in this manner are large enough to be observed or negligible. Thus, it remains poorly understood how LFPs are generated in these subcortical structures without layers [14,15].

Here, we test a hypothesis of LFP generation in the non-laminar brain regions by inhomogeneous excitation [1]. We hypothesized that a sink and a source are formed between the dendrites of the neurons at the periphery of a focal excitatory input in the striatum, although they are canceled out in the middle (Fig 1C). In order to test the biological plausibility of the hypothesis, we performed the simulation of LFP generation in the striatum using multicompartment model and compared it with that in the cortex. We also examined simplified models of the LFP generation in the striatum to verify the hypothesis. We propose the models as a tool to interpret LFPs in the non-laminar brain regions.

## Methods

Simulations were performed on NEURON 7.0 [16,17] and LFPy 1.1.3 [18]. LFPs were obtained by the linear summation of the electric potentials generated by single neuron models. The extracellular conductivity was set to *σ* = 0.3 S/m [12,19,20]. The simulation time resolution was set to 0.0625 ms. The source code of the simulations is available from http://modeldb.yale.edu/258844.

To simulate the LFPs in the cerebral cortex, we used a model of layer-5 pyramidal cell from cat visual cortex [21] downloaded from ModelDB (https://modeldb.yale.edu/2488). Sixty-four recording electrodes were placed from the point (0 μm, 0 μm, –1500 μm) (denoting *x*-, *y*-, and *z*-coordinates, respectively, unless otherwise stated) to another (0 μm, 0 μm, 500 μm) at the regular intervals (31.7 μm). The somata of pyramidal neurons were randomly distributed with the density of 60 000/mm^3^ in a cube centered at the point (0 μm, 0 μm, –800 μm) with a side length of 600 μm [22]. Pyramidal neuron models were randomly rotated around the *z*-axis. For each pyramidal neuron, we randomly distributed 200 AMPA synapses on apical dendrites and discarded those which were outside the sphere with a radius of 150 μm centered at the point (0 μm, 0 μm, 0 μm). We randomly distributed 20 GABA_A_ synapses on basal dendrites of each pyramidal neuron. We simulated the LFP generated by 12 960 pyramidal neurons, 80 750 AMPA synapses, and 259 200 GABA_A_ synapses. Pyramidal neuron models were passive with the following parameter values: resting membrane potential *V*_*E*_ = −65 mV, membrane resistivity *R*_*m*_ = 30 kΩ cm^2^, axial resistivity *R*_*i*_ = 150 Ω cm, and membrane capacitance *C*_*m*_ = 1 μF cm^-2^ [12,21].

To simulate the LFPs in the striatum, we used a medium-sized spiny neuron (MSN) model from mouse striatum [23] downloaded from ModelDB (https://modeldb.yale.edu/151458). Sixty-four recording electrodes were placed from the point (0 μm, 0 μm, –500 μm) to another (0 μm, 0 μm, 500 μm) at regular intervals (15.9 μm). The somata of MSN were randomly distributed with the density of 100 000/mm^3^ in a cube centered at the point (0 μm, 0 μm, –800 μm) with a side length of 600 μm [24,25]. MSN models were randomly rotated around the somata. For each MSN, we randomly distributed 80 AMPA (120 GABA_A_) synapses on dendrites and discarded those which were outside the sphere centered at the point (0 μm, 0 μm, 0 μm) with a radius of 150 μm (200 μm). Discarding MSNs without synapses, we simulated the LFP generated by 9182 MSNs with 112 987 AMPA synapses and 405 655 GABA_A_ synapses. MSN models were passive with the following parameter values: resting membrane potential *V*_*E*_ = −70 mV, membrane resistivity *R*_*m*_ = 45.2 kΩ cm^2^, axial resistivity *R*_*i*_ = 100 Ω cm, and membrane capacitance *C*_*m*_ = 1 μF cm^-2^ [23]. The membrane resistivity of the middle components [23] was used.

**Fig 2:**
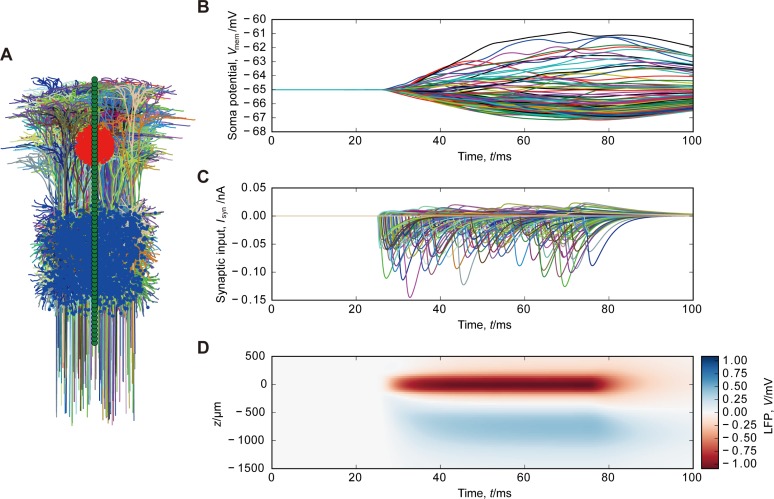
Simulation of LFPs generated by cortical pyramidal neurons. (A) Spatial arrangement of pyramidal neurons and synapses. Dendrites of each pyramidal neuron are indicated by a color. AMPA and GABA_A_ synapses are represented by black and blue dots, respectively. Green dots indicate the recording electrodes. The topmost and bottommost electrodes are positioned at 500 μm above and 1500 μm below the mass center of AMPA synapses, respectively. One in 100 pyramidal neurons is plotted. (B) Somatic membrane potentials of pyramidal neurons. (C) Synaptic currents. (D) Simulated LFP.

**Fig 3:**
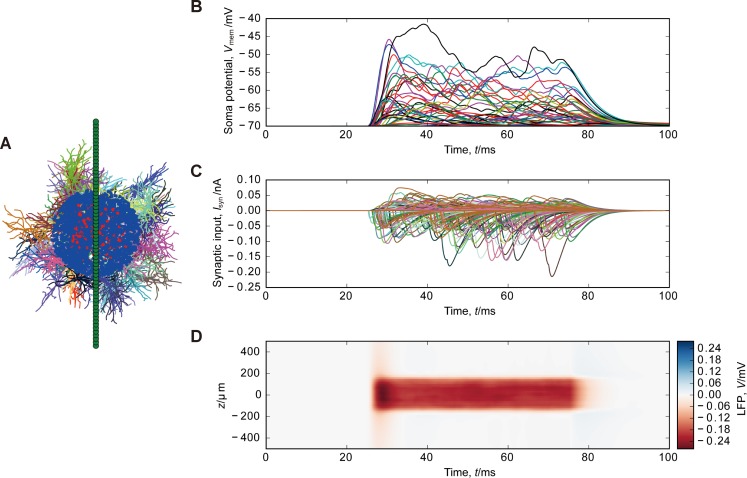
Simulation of LFPs generated by MSNs. (A) Spatial arrangement of MSNs and synapses. Dendrites of each MSNs are indicated by a color. AMPA and GABA_A_ synapses are represented by black and blue dots, respectively. Green dots indicate the recording electrodes. The topmost and bottommost electrodes are positioned at 500 μm above and 500 μm below the mass center of the somata, respectively. One in 100 MSNs is plotted. (B) Somatic membrane potentials of MSNs. (C) Synaptic currents. (D) Simulated LFP.

**Fig 4:**
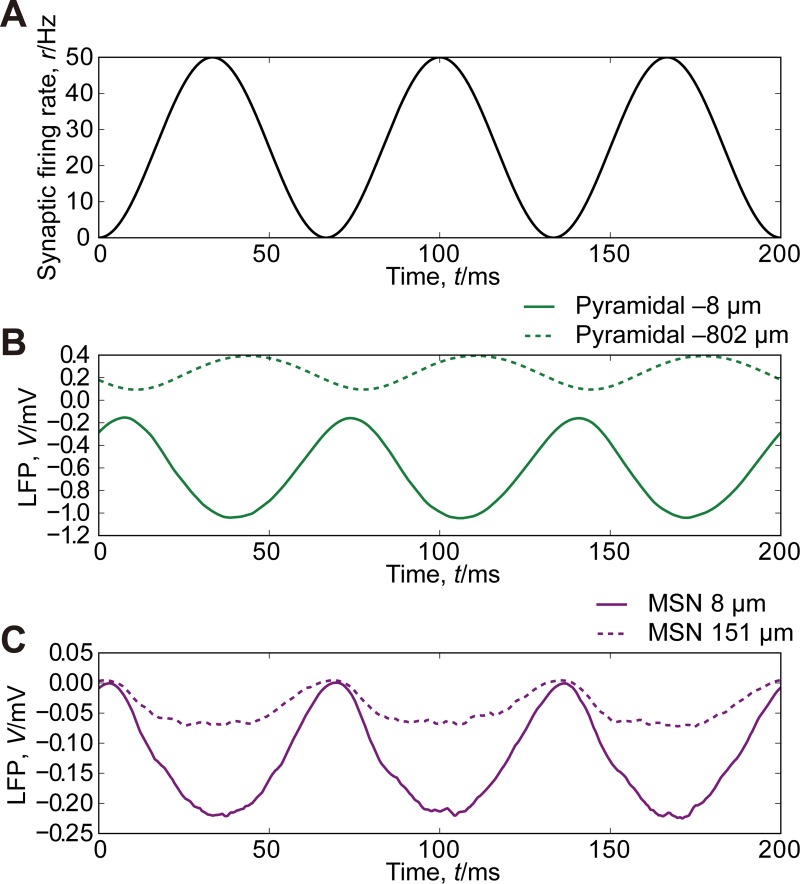
Simulation of LFPs generated by fluctuating synaptic inputs. (A) The time course of the rate of synaptic activation. (B) LFPs generated by pyramidal neurons at 8 μm below (solid line) and 802 μm below (dashed line) the mass center of AMPA synapses. (C) LFPs generated by MSNs at 8 μm above (solid line) and 151 μm above (dashed line) the mass center of AMPA synapses.

The reversal potential and the rise and decay time constants of the AMPA receptors were set to 0 mV, 1 ms, and 3 ms, respectively; the reversal potential, the rise and decay time constants of the GABA_A_ receptor were set to –70 mV, 1 ms, and 12 ms, respectively. Synaptic conductances were set to 1 nS. In Figs 2 and 3, synapses were activated with inter-spike intervals obeying a gamma distribution with the shape parameter *k* = 0.5 and the scale parameter *θ* = 40 ms during the interval from 25 ms to 75 ms. In Fig 4, the synaptic activation followed a nonstationary Poisson process with intensity function *r*(*t*) = 25(1−cos *ωt*) Hz, where $\omega =\frac{2\pi \times 15}{1000}{\mathrm{ms}}^{-1}$.

## Results

**Fig 5:**
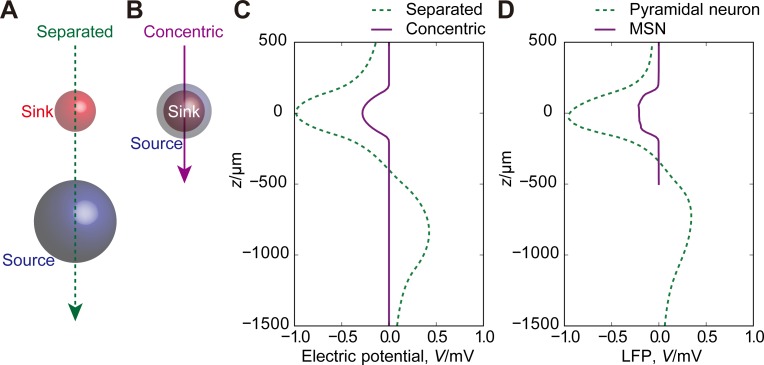
Simplified models of LFPs. (A,B) Schematic representation of separated (A) and concentric (B) sink and source in the simplified models. (C) LFPs generated by the simplified models. (D) Average of LFPs over the interval from 25 ms to 75 ms for the simulations presented in Figs 2 and 3.

Our hypothesis is that focal excitatory input generates a current sink surrounded by a concentric current source and that these current sink and source enable us to observe LFPs even in nuclei without laminar structures. To validate this hypothesis, we performed the simulations of conductance-based models. First, we simulated LFPs in the cerebral cortex to examine whether the size of the observed LFP falls in the experimentally reported range [1,9]. Pyramidal neurons were randomly distributed with a density of 60 neurons in a cube with a side length of 100 μm [22] (Fig 2A). Excitatory synapses were distributed on the apical dendritic segments in a sphere with a radius of 150 μm, and inhibitory synapses were distributed on basal dendrites. We simulated the synaptic currents and membrane potentials assuming that synapses are activated at 50 Hz (Fig 2B and 2C). Electrodes recorded LFPs of –0.95 mV at the apical dendrites and 0.33 mV at the basal dendrites (Figs 2D and 5D), which are on the order of experimentally reported values [1,9].

Second, we simulated LFPs generated by MSNs in the striatum. MSNs were randomly distributed with a density of 100 neurons in a cube with a side length of 100 μm [24,25] (Fig 3A). Excitatory and inhibitory synapses were distributed on the dendritic segments in the spheres with radii 150 μm and 200 μm. Fig 3B and 3C show the membrane potentials and synaptic currents. The same synaptic parameter values as in pyramidal neurons were used. The numerical simulation gave the LFP of –0.23 mV at the mass center of the somata (Figs 3D and 5D). This is approximately one-fourth of the LFP in the cortex and consistent with the experimentally observed LFP size [4].

Third, we examined LFPs generated by fluctuating synaptic inputs. Fig 4A shows the time course of the synaptic firing rate, which is an oscillation in the beta band. The maximal firing rate is 50 Hz, which is identical to the synaptic firing rates in Figs 2 and 3. The amplitudes of LFPs exhibited by pyramidal neurons (Fig 4B) and MSNs (Fig 4C) were within 20% of those in the simulations without fluctuation (Figs 2D and 3D). The standard deviations of LFPs at the electrode closest to the mass center of AMPA synapses were 0.31 mV for pyramidal neurons and 0.075 mV for MSNs.

Last, in order to obtain a simpler picture of LFP generation, we radically simplified the above-mentioned model consisting of vast number of neurons and synapses into a spherical current sink and a source and examined whether this simplified model can reproduce LFP profiles. In the cerebral cortex, excitatory synaptic activity induces a current sink on the apical dendrites of pyramidal neurons; a current source is found on basal dendrites. We can model this by a spherical current sink at the point (0 μm, 0 μm, 0 μm) with a radius of 150 μm and a source at the point (0 μm, 0 μm, –800 μm) with a radius of 300 μm (Fig 5A, “separated model”). The electric potential at the point (0 μm, 0 μm, *z*) is given by

$$-V(z,200\;\mu m,i_{0})+V(z+800\;\mu m,300\;\mu m,{\left(150/300\right)}^{3}i_{0})$$

(Fig 5C, dotted line), where *V*(*d*,*r*,*i*) is the electric potential at the point (0 μm, 0 μm, *d*) arising from the spherical current source centered at the origin with radius *r* whose current density is *i*. *V*(*d*,*r*,*i*) is defined by

$$V(d,r,i)=\left\{ \begin{array}{c} \frac{i(3r^{2}-d^{2})}{6\sigma }\;|d|\leq r\\ \;\frac{ir^{3}}{3|d|\sigma }\;|d|>r \end{array}\right..$$

Here we set *i*_0_ = 3.0×10^−5^ nA μ*m*^−3^.

In the striatum, although excitatory input is not localized to a specific part of MSN dendrites, focal excitatory input induces a current sink. If the focal excitation covers only a part of dendrites of an MSN, the other part becomes a current source (Fig 1C). This can be represented by a spherical current sink at (0 μm, 0 μm, 0 μm) with a radius of 150 μm and a source at (0 μm, 0 μm, 0 μm) with a radius of 200 μm (Fig 5B, “concentric model”). The electric potential at (0 μm, 0 μm, *z*) is given by

$$-V(z,150\;\mu m,i_{0})+V(z,200\;\mu m,{\left(150/200\right)}^{3}i_{0})$$

(Fig 5C, solid line). The concentric model yields a smaller LFP than the separated model. The most remarkable difference is that the separated model takes on both signs but the concentric model takes only on the negative sign. Both simplified models agreed quite well with the simulation results of conductance-based models (Fig 5C and 5D).

## Discussion

The present study examined the hypothesis that, in nuclei without laminar structures, focal excitatory input induces a concentric current sink and source that generate LFPs. Both the simulations with multicompartment models and the analysis of simplified models showed that focal excitatory input into non-laminar nuclei can induce a concentric sink–source structure, which generates LFPs with approximately one-fourth of the amplitude of LFPs observed in the laminar cortex.

A sizable contribution to LFP has been believed to be made only by the laminar organization of pyramidal neurons because they can generate strong current dipoles owing to spatially separated current sinks and sources on apical and basal dendrites [1,12,13]. In contrast, spherically symmetric neurons such as MSNs have been believed to give rise to so small dipoles that they cannot generate observable LFPs. However, these arguments did not take into account the effect of inhomogeneous excitatory input [1]. The present study showed that focal excitatory input, which is the simplest form of inhomogeneous excitatory inputs, can generate LFPs on the order of 0.1 mV in regions without laminar organization. The present results also suggest that the contribution of layer-4 stellate neurons of the cortex to the cortical LFPs should not be ignored.

LFPs have been experimentally reported in the striatum [2–5] and the thalamus [6–9]. However, how to interpret LFPs in these regions has been unclear [14]. Some authors argue that at least part of LFPs recorded in the striatum could be accounted for by volume conduction from a distant structure [26,27]. The present study does not exclude the contribution of volume conduction to subcortical LFPs. For instance, the synchronized firing of the pyramidal neurons in all cortical areas forms the electric gradient only at the boundary between the sheets of the current sink and source in the superficial and deep cortical layers, respectively or vice versa, and the subcortical structures can thus show LFPs of roughly the same magnitude and polarity as the deep cortical layers (Fig 1D). On the other hand, there have been reports of subcortical LFPs that cannot be attributed to volume conductance alone. For instance, LFPs recorded from human subthalamic nucleus showed clearly local heterogeneity within [28]. LFPs recorded in the subcortical structures can be the superposition of the potentials depicted in Fig 1C and 1D. The present results provide a theoretical explanation to these experimental findings and suggest that LFPs are an indicator of focal excitatory input in these subcortical regions. Hence, the recording of LFPs with multielectrode arrays allows us to identify the functional localization of focal excitatory input, for instance, glutamatergic cortical afferents in the striatum and the thalamic nuclei. Estimating the spatial and temporal scale of focal excitatory input will facilitate the physiological understanding of these subcortical regions. As can be seen from Fig 5C and 5D, LFPs predicted by spherical current sinks and sources closely approximate those obtained by the simulation of conductance-based models. This allows us to estimate the location of putative focal excitatory input by fitting the experimental LFP data with the electric potentials that are predicted from simple spherical current sinks and sources. Although replacing uniform spherical current sink and source distributions with other distributions, such as the Gaussian distribution, might give a better fit to data, it is expected to provide qualitatively the same results.
